# A disrupted transsulphuration pathway results in accumulation of redox metabolites and induction of gametocytogenesis in malaria

**DOI:** 10.1038/srep40213

**Published:** 2017-01-16

**Authors:** Divya Beri, Balu Balan, Shweta Chaubey, Suraj Subramaniam, Bachu Surendra, Utpal Tatu

**Affiliations:** 1 Department of Biochemistry, Indian Institute of Science, Bangalore, 560012, India

## Abstract

Intra-erythrocytic growth of malaria parasite is known to induce redox stress. In addition to haem degradation which generates reactive oxygen species (ROS), the parasite is also thought to efflux redox active homocysteine. To understand the basis underlying accumulation of homocysteine, we have examined the transsulphuration (TS) pathway in the parasite, which is known to convert homocysteine to cysteine in higher eukaryotes. Our bioinformatic analysis revealed absence of key enzymes in the biosynthesis of cysteine namely cystathionine-β-synthase and cystathionine-γ-lyase in the parasite. Using mass spectrometry, we confirmed the absence of cystathionine, which is formed by enzymatic conversion of homocysteine thereby confirming truncation of TS pathway. We also quantitated levels of glutathione and homocysteine in infected erythrocytes and its spent medium. Our results showed increase in levels of these metabolites intracellularly and in culture supernatants. Our results provide a mechanistic basis for the long-known occurrence of hyperhomocysteinemia in malaria. Most importantly we find that homocysteine induces the transcription factor implicated in gametocytogenesis namely AP2-G and consequently triggers sexual stage conversion. We confirmed this observation both *in vitro* using *Plasmodium falciparum* cultures, and *in vivo* in the mouse model of malaria. Our study implicates homocysteine as a potential physiological trigger of gametocytogenesis.

The malaria parasite *Plasmodium falciparum* is an auxotroph for several amino acids^1^. It heavily depends on host haemoglobin degradation to manage its amino acid supplies. Haem degradation is known to generate an enormous amount of ROS^2^,^3^. This along with the host’s immune system poses a substantial redox stress on the intraerythrocytic development of the parasite. An efficient redox balance system is, thus, a necessity for *Plasmodium*.

The redox biology of *Plasmodium* is relatively ill explored. On one hand it is armed with robust superoxide dismutase (SOD), thioredoxin and glutathione redox system^4^,^5^,^6^, but on the other hand it lacks canonical unfolded protein response (UPR) components^7^, catalase and the classical glutathione peroxidase^8^,^9^. Also, its repertoire of protein disulphide isomerase (PDI), thioredoxin and glutaredoxin is limited as compared to its other eukaryotic counterparts^10^,^11^. Despite living in an environment that is prone to redox perturbation, the parasite seems to be ill equipped with redox stress combating machinery. The parasite does possess the common cellular redox buffer glutathione (GSH), however, its precursor amino acid cysteine can only be obtained from the host.

While searching for mechanisms used by the parasite to combat this redox stress, we found that the model redox perturbant namely DTT triggers sexual stage transition in the malaria parasite^7^. In the current study, we provide the physiological relevance of this observation. We show that redox perturbation caused due to the intracellular growth of the parasite results in accumulation of redox metabolites, which in turn may be responsible for triggering gametocytogenesis. Using bioinformatics and metabolomics approach together with *in vitro* and *in vivo* gametocytogenesis assays we show that the absence of key enzymes in the transsulfuration pathway contribute to the accumulation of redox metabolites such as homocysteine, which trigger the pathway for sexual stage conversion. Our results suggest that self-generated redox stress, the lack of mechanisms to cope up with the redox perturbation and the ability of accumulated redox metabolites to trigger gametocytogenesis, together provide for an “autocrine” regulation of sexual stage conversion in the malaria parasite.

## Results

### Bioinformatics analysis reveals a disrupted transsulphuration pathway in the malaria parasite

Previous studies have reported accumulation of the redox metabolite homocysteine in malaria infected patients^12^. It has been shown that levels of homocysteine in the plasma positively correlate with parasitemia in the patient. To understand the mechanisms underlying hyperhomocysteinemia in malaria, we looked at the transsulphuration (TS) pathway which involves homocysteine as an important intermediate. It is a well-studied pathway in higher eukaryotes but little is known about it in the malaria parasite^13^. TS pathway is the metabolic bridge between the methionine cycle and cysteine. In the TS pathway cystathionine β synthase (CBS) catalyzes the first step wherein homocysteine and serine condense to form cystathionine which is further converted to cysteine by the enzyme cystathionine γ lyase (CGL)^14^. To understand whether a complete TS pathway is operating in *Plasmodium*, we performed a BLASTP analysis using CBS and CGL sequences of *Saccharomyces cerevisiae* as the template as this organism possesses a complete TS pathway. The representative protein sequences of CBS (YGR155W) and CGL (YAL012W) of *Saccharomyces cerevisiae* were retrieved from FungiDB keeping the threshold of E value cutoff as 0.01. The presence of specific catalytic domains of these enzymes in other proteins was verified using a CD search.

**Figure 1:**
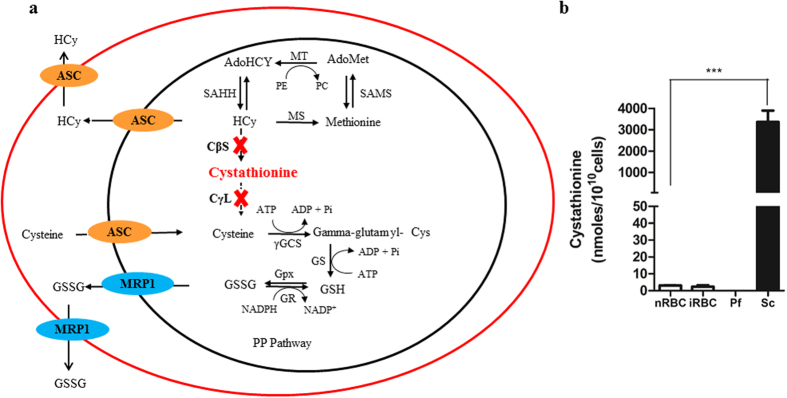
(**a**) A schematic representation of a truncated transsulphuration pathway in malaria parasite: Absence of CBS and CGL enzymes results in a truncated transsulphuration (TS) pathway in the parasite. Abbreviations: CβS-Cystathionine beta synthase, CγL- Cystathionine gamma lyase, MT- Methyl Transferase, MS- Methionine Synthase, SAHH- S-Adenyl -L- Homocysteine Hydrolase, SAMS- S-Adenosyl Methionine Synthetase, γGCS- Gamma glutamyl Cysteine Synthetase, GS- Glutathione Synthetase, GR- Glutathione Reductase, GPx- Glutathione Peroxidase, PE- Phospho ethanolamine, PC- Phosphatidyl choline, HCy- Homocysteine, AdoMet- S-Adenosyl Methionine, AdoHCy- S-Adenyl-L- Homocysteine, GSH- Reduced Glutathione, GSSG- Oxidized Glutathione, MRP1- Multi Drug Resistance Protein1, ASC- Amino Acid Transporter Family. (**b**) Absence of cystathionine confirms a disrupted TS pathway in *Plasmodium falciparum*. The figure shows levels of cystathionine in normal RBC (nRBC), infected RBC (iRBC), *Plasmodium falciparum* (Pf) and *Saccharomyces* cerevisiae (Sc) as measured by LC-MS. Equal number of cells from each were analysed using LC-MS approach. *Plasmodium falciparum*, nRBC and iRBC showed negligible levels of cystathionine while *Saccharomyces* cerevisiae, the eukaryotic positive control for the transsulphuration pathway, showed a significant level of this metabolic intermediate. Cystathionine is a key metabolic intermediate of the TS pathway and its absence in *Plasmodium* validates the truncation of the TS pathway in the parasite (P = 0.0002, n = 3).

Our results show that the genes coding for CBS and CGL are missing in *Plasmodium* species. Absence of these enzymes would therefore result in accumulation of homocysteine in the parasite. With this information, we developed a model (Fig. 1a) showing the probable fate of accumulated homocysteine in the parasite. Our results suggest the possibility that the parasite may efflux homocysteine into the RBC. The RBC possess the methionine cycle but also lack the TS pathway. Thus, they can form homocysteine but cannot convert homocysteine to cysteine^15^,^16^,^17^. The RBC may further expel the accumulated homocysteine out into the plasma where it is known to concentrate during malaria infection^12^. This observation correlates well with the fact that malaria patients exhibit high levels of homocysteine in their plasma and it appears that efflux of accumulated homocysteine into the infected RBCs and subsequently to the plasma may be the cause underlying hyperhomocysteinemia in malaria patients.

### Metabolic analysis confirms the truncation of the transsulphuration pathway

Our bioinformatics analysis pointed out absence of key metabolic enzymes which are involved in catalysis of homocysteine to cysteine in the transsulfuration pathway. To validate our analysis, we resorted to LC-MS to sensitively analyse the presence of cystathionine which is a key intermediate in the TS pathway. Absence of CBS would preclude synthesis of cystathionine in the malaria parasite. *Saccharomyces cerevisiae* has a fully functional TS pathway^18^,^19^,^20^ and therefore served as our positive control for the presence of cystathionine. Towards this, infected RBCs (iRBC) were selectively enriched in late trophozoite and schizont stage. Saponin lysis was used to separate the RBC (saponin lysate) and the parasite compartment (saponin pellet) which was further lysed in hypotonic condition. As a control, normal RBCs (nRBC) were identically treated. *Saccharomyces cerevisiae* was lysed by glass bead lysis. Saponin lysate (both from nRBC and iRBC), saponin pellet and yeast lysate were further processed as described under Methods and were analysed for the presence of cystathionine by LC-MS. Data obtained was normalized to 10^10^ cells in RBC, parasite and yeast.

*Saccharomyces cerevisiae* possesses a full complement of enzymes of the transsulphuration pathway^19^. On the basis of this, we reasoned that *Saccharomyces* should have detectable levels of the key intermediate cystathionine while RBCs and parasite having a disrupted TS pathway should not have cystathionine. As expected, *Saccharomyces* lysate had 3000 nmoles/10^10^cells cystathionine (Fig. 1b). On the other hand, RBCs and *Plasmodium* did not show detectable levels of cystathionine. This strengthens our bioinformatics analysis suggesting absence of enzymes mediating the formation (by CBS) and breakdown of cystathionine (by CGL) in the TS pathway. Thus, the parasite appears to lack the pathway which would detoxify the redox active homocysteine resulting in its accumulation and efflux.

### Quantification of redox metabolites in the host erythrocytes and spent medium on *Plasmodium* infection

As described previously, the parasite lacks the enzymes required to convert homocysteine to cysteine. This would imply that the parasite may accumulate and efflux out excess homocysteine into the plasma of the infected patient^12^. Indeed, previous studies have hinted at increase in homocysteine levels in the serum of malaria patients Also, attempts have been made to quantify levels of glutathione in the host RBCs and the spent medium. These studies have relied on conventional methods for measuring these metabolites and there is lack of agreement among different reports about the absolute values and the form of glutathione being effluxed^2^,^21^,^22^,^23^. We, therefore, resorted to the use of mass spectrometry (MS) based quantification of homocysteine (HCy), reduced glutathione (GSH) and oxidized glutathione (GSSG) intracellularly as well as in the spent medium of *P. falciparum* infected RBC (iRBC). For measurement of the redox metabolites intracellularly we used an approach of Percoll purification and saponin lysis. Using 60% Percoll, we enriched RBCs infected with late trophozoites and schizonts (>95%). This was followed by saponin lysis to differentiate between the metabolite content within the parasite (saponin pellet) and the infected host cell compartment (saponin lysate). The spent medium was collected after incubating iRBCs at a 5% hematocrit and parasitemia ranging between 3–4.7% in cRPMI for 24 hours and processed for MS anaylsis as described under Methods. Correction for parasitemia was performed (as described under Methods) to calculate efflux from the iRBCs. Using identical conditions, spent medium as well as intracellular content of normal RBC (nRBC) were collected to serve as control.

**Figure 2:**
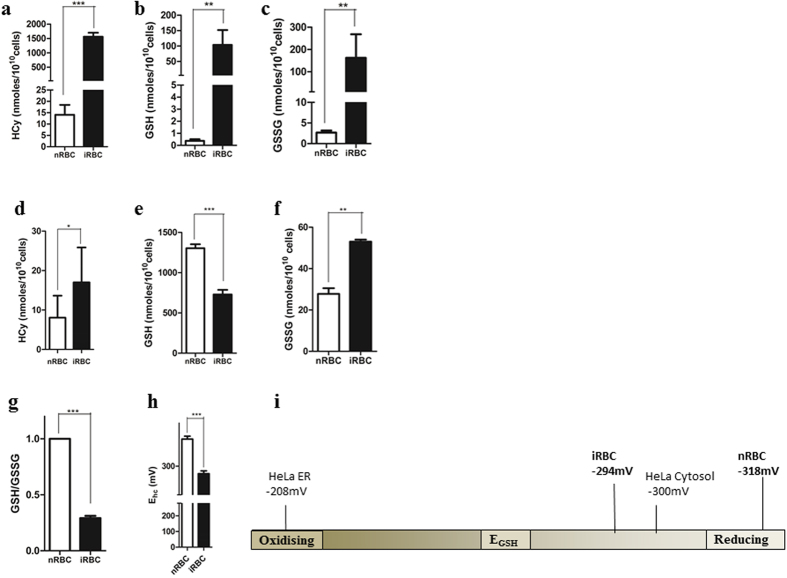
Accumulation and efflux of redox metabolites in *Plasmodium*-infected erythrocytes and spent medium. Levels of reduced glutathione (GSH), oxidized glutathione (GSSG) and homocysteine were measured in the spent medium and erythrocytes infected with *Plasmodium* (**a,b,c**). represents efflux of metabolites as measured in the spent medium of *Plasmodium*-infected RBCs (iRBC) and an equal number of normal RBC (nRBC) (**a**) Elevated homocysteine efflux from iRBC compared to nRBC (P < 0.0001, n = 5) (**b**) increased GSH efflux from iRBCs compared to nRBC. (P = 0.0013, n = 7) (**c**) Increased GSSG efflux from iRBCs compared to nRBC. (P = 0.0069, n = 7) (**d**,**e**,**f**). represents measurement of metabolites in the saponin lysate from nRBC and iRBC. (**d**) A two-fold increase of homocysteine in iRBC compared to nRBC was detected. (P = 0.0141, n = 4). (**e**) A 1.7-fold decrease in GSH levels in iRBC compared to nRBC was detected. (P = 0.0002, n = 3) (**f**) a 2-fold increase in GSSG levels in infected RBCs compared to normal RBCs was detected. (P = 0.0059, n = 3). (**g**) Figure showing a 3.7-fold decrease in GSH/GSSG ratio in iRBC compared to nRBC (P = 0.0090, n = 3). (**h**) Bar graph depicting a drop in half cell reduction potential value (Eh) on *Plasmodium* infection (p < 0.0001, n = 3) (**i**) Redox scale showing the shift of half-cell reduction potential of infected RBCs towards the oxidizing end by ~25 mV compared to uninfected RBCs.

As shown in Fig. 2a,b and c, we found significantly higher levels of HCy, GSH as well as GSSG in the spent medium of the iRBC as compared to nRBCs. HCy levels were measured to be 1560 ± 62.16 nmoles/10^10^ cells in iRBC compared to 14.06 ± 1.952 nmoles/10^10^ cells in nRBCs. Both forms of glutathione were effluxed: GSH efflux was 103.7 ± 18.15 nmoles/10^10^ cells in iRBCs compared to 0.3731 ± 0.0516 nmoles/10^10^ cells in nRBCs while GSSG efflux was measured to be 162.4 ± 39.82 nmoles/10^10^ cells in iRBCs compared to 2.657 ± 0.1997 nmoles/10^10^ cells in nRBCs. Our results suggest an efflux of redox metabolites from iRBC to the medium (iMed) during the growth of malaria parasite in culture.

We also observed an increase in the intracellular levels of homocysteine in the iRBC as compared to nRBC (Fig. 2d). While GSSG levels showed a similar increase in the host cell (53 ± 0.5774 nmoles/10^10^ cells in iRBC compared to 27.76 ± 1.607 in nRBC), GSH levels decreased (729 ± 33.45 nmoles/10^10^ cells in iRBC compared to nRBC (1304 ± 28.15 nmoles/10^10^ cells) (Fig. 2e and f). A similar decrease in GSH levels has been reported by other groups^2^,^21^,^22^.

Using the values of GSH and GSSG obtained by our MS analysis of the host compartment, we found a 3.7-fold decrease in the GSH: GSSG ratio in the iRBC compared to nRBC (Fig. 2g). The ability to shift the redox homeostasis of the host is shared by many other viruses, bacteria and protozoa. As shown in Table 1, many infections caused by intracellular pathogens result in a 2-4-fold decrease in the GSH: GSSG ratio of the host cell^2^,^21^,^22^,^23^,^24^,^25^,^26^,^27^,^28^,^29^,^30^,^31^,^32^,^33^. Importantly, this altered redox state is often utilized by the pathogen to facilitate its pathogenesis and/or transmission.

The measurement of GSH and GSSG levels in the host cell cytoplasm also allowed us to estimate the reduction potential of the normal and infected erythrocytes^34^. The glutathione redox couple (GSH/GSSG) is the most abundant redox modulating buffer in the cell and changes in the half-cell reduction potential of the GSSG/2GSH couple are known to correlate with biological status of the cell^35^,^36^. From the absolute values of GSH and GSSG, we estimated the redox potential of the GSSG/2GSH redox couple using the Nernst equation (see Methods). Figure 2i represents the redox scale ranging from −200mV to −320 mV. HeLa cell ER E_GSH_ value on the extreme oxidizing end (−208 mV) and HeLa cell cytosolic E_GSH_ (−300 mV) at the reducing end^37^,^38^. As shown in Fig. 2h, the E_GSH_ value of the erythrocyte increases by about 25 mV (from −318 in uninfected erythrocytes to −294 in parasitized erythrocytes. This suggests that the parasitized erythrocyte becomes mildly oxidizing upon infection. Our study provides the first comprehensive MS-based analysis of redox status of the *Plasmodium-*infected erythrocytes and its spent medium.

To assess the overall status of the transsulphuration pathway, we also measured related metabolites involved in the pathway, namely, methionine (Met), S-adenosyl methionine (SAM), S-adenosyl homocysteine (SAH) and cysteine (Cys) in nRBC, iRBC, nMed and iMed (Fig. S1a and b). The level of the essential amino acids, methionine and cysteine, decreased in the iMed (Met: 1476 ± 265.5 nmoles/10^10^ cells; Cys: 1064 ± 279.2 nmoles/10^10^ cells) compared to the nMed (Met: 1867 ± 236.7 nmoles/10^10^ cells; Cys: 2592 ± 33.28 nmoles/10^10^ cells). While Met decreased in infected cells (14.94 ± 1.048 nmoles/10^10^ cells in the iRBC compared to 142 ± 0.7335 nmoles/10^10^ cells in nRBC), Cys levels did not show much change (2652 ± 50.60 nmoles/10^10^ cells in iRBC compared to 2341 ± 23.93 nmoles/10^10^ cells in nRBC). A similar trend was observed for SAM and SAH. Its levels increased in the iMed (SAM: 1.048 ± 0.01958 nmoles/10^10^ cells; SAH: 0.9383 ± 0.1205 nmoles/10^10^ cells) and iRBC (SAM: 8.992 ± 0.8553 nmoles/10^10^ cells; SAH: 6.783 ± 0.8332 nmoles/10^10^ cells) compared to nMed (SAM: 0.08713 ± 0.003494 nmoles/10^10^ cells; SAH: 0.2006 ± 0.02488 nmoles/10^10^ cells) and nRBC (SAM: 4.643 ± 0.4742 nmoles/10^10^ cells; SAH: 5.937 ± 0.6817 nmoles/10^10 ^cells), respectively. Above results have been tabulated in Table 2.

### Homocysteine accumulation induces gametocytogenesis

**Figure 3:**
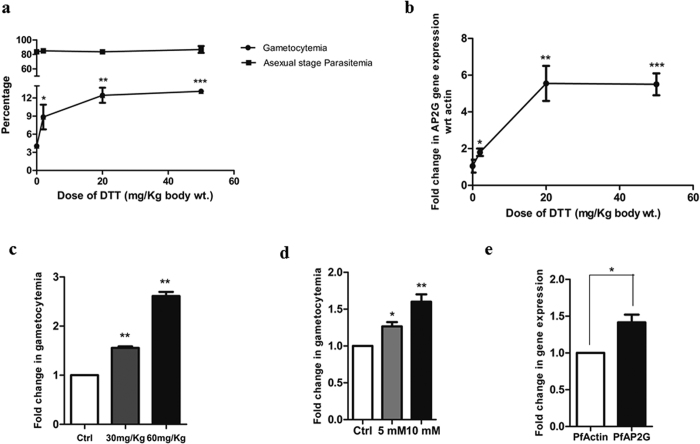
Redox metabolite induces gametocytogenesis in malaria parasite. (**a**,**b**) Represents *in vivo* induction of gametocytemia on DTT treatment. Mice infected with *P. berghei* were injected with DTT at concentrations 2, 20 and 50 mg/kg and gametocytemia was scored microscopically after 48 h hrs. (**a**) Dose-dependent increase in gametocytemia in mice injected with DTT (n = 4 in each set) compared to vehicle-treated mice. At 2 mg/kg, percentage gametocytemia was 9.167 ± 2.581(P = 0.0263, n = 3). At 20 mg/kg, percentage gametocytemia was 12.7 ± 1.767 (P = 0.0010, n = 3) and at 50 mg/kg. The percentage gametocytemia did not show further significant increase (at 50 mg/kg, percentage gametocytemia was 13.13 ± 0.40; P < 0.0001, n = 3) with increase in DTT dose. The asexual parasitemia remained similar at all doses. (**b**) qRT-PCR analysis showing fold change in AP2-G expression. The expression of AP2-G increased upto 20 mg/kg (6-folds) and then no further increase was observed with increase in DTT dosage. (**c**,**d**,**e**) represents induction of gametocytogenesis on homocysteine treatment (**c**) Mice infected with *P. berghei* were injected with homocysteine at concentrations 30 and 60 mg/kg (n = 4) and gametocytemia was scored microscopically after 48 hrs. Dose-dependent increase was seen with a 1.5-fold increase in the 30 mg/kg group (P = 0.026, n = 3) and a 2-fold increase in 60 mg/kg group (P = 0.029, n = 3) compared to vehicle treated mice.(**d**) *P. falciparum* cultures were treated with homocysteine at a concentration of 5 mM and 10 mM for 1 hr and gametocytemia was calculated on the fifth day. At a concentration of 5 mM, a 1.3-fold induction of gametocytemia was observed (P = 0.0153, n = 3). At 10 mM a 1.5-fold induction of gametocytemia was observed (P = 0.0091, n = 3). (**e**) *P. falciparum* cultures were treated with 5 mM homocysteine for 1 hr. qRT-PCR analysis showing a 1.5-fold upregulation of AP2-G expression with respect to actin (P = 0.0202, n = 3).

In our previous study we have shown that DTT, which is commonly used as a model redox perturbant, is able to trigger gametocytogenesis in the malaria parasite^7^. While the previous study examined the effect of DTT only *in vitro* using cultured *P. falciparum*, here we have examined the effects of DTT in an *in vivo* mouse model of malaria using *P. berghei.* We examined the effects of an increasing concentrations of DTT on gametocytogenesis. 2, 20 or 50 mg/kg of DTT were injected intraperitoneally in *P. berghei* infected mice as described under Methods. Vehicle treated, infected mice served as controls. Parasitemia and gametocytemia were monitored microscopically after 48 hrs of DTT injection. As shown in Fig. 3a, 2 mg/kg DTT injection showed a 2-fold increase in gametocytemia as compared to the vehicle treated controls. At 20 mg/kg, we observed a 4-fold increase with ~13% gametocytemia. DTT induced gametocytemia reached saturation at 20 mg/kg of DTT. We also monitored the levels of AP2G gene expression from the same samples using RT-PCR as described under methods. As shown in Fig. 3b, a similar trend was observed in the AP2-G levels. 2 mg/kg DTT showed a 2- fold increase in AP2-G expression. At 20 mg/kg DTT and 50 mg/kg DTT doses, a maximum of 6-fold induction of AP2-G was observed. These results provided *in vivo* validation of our previous observations made using *in vitro* cultures of *P. falciparum.*

Homocysteine is a well-known redox perturbant and hyperhomocystenemia is known to be associated with redox stress in eukaryotes. Given that homocysteine levels increase in *Plasmodium* infected erythrocytes, we asked if high homocysteine could play a role in inducing gametocytogenesis. We performed this experiment both *in vitro* and *in vivo* by treating *P. falciparum* cultures with 5 & 10 mM homocysteine for 1 h and by injecting 30 and 60 mg/Kg homocysteine in *P. berghei* infected mouse. Gametocytes were counted after 5 days in case of *P. falciparum* and after 48 h in case of *P. berghei.* As shown in Fig. 3d, a 1.5-fold increase in the number of gametocytes was observed upon homocysteine treatment *in vitro* and the effect was more pronounced *in vivo* with more than 2-folds induction observed on injection of 60 mg/kg of homocysteine (Fig. 3c). The result was further supported by the observation that there was an increase in expression of AP2-G in *P. falciparum* upon homocysteine treatment. A 1.5-fold induction of AP2-G was seen on 5 mM homocysteine treatment for one hour (Fig. 3e). As is evident, the effect was more pronounced with DTT as compared to homocysteine. This could be because DTT is a stronger reducing agent with two active thiols as compared to homocysteine and thus would lead to greater redox perturbation. Overall these results suggested that homocysteine accumulating due to the absence of CBS and CBL enzymes in *Plasmodium* is able to induce gametocytogenesis in *P. falciparum* as well as *P. berghei*. Our results, for the first time, point to a potential physiological trigger of gametocytogenesis in malaria.

### Homocysteine pre-exposure triggers enhanced gametocytogenesis

With increasing asexual cycles, the levels of homocysteine effluxed would also increase as there would be more number of parasitized RBCs that would be effluxing out homocysteine. This would be taken up by neighbouring erythrocytes as RBCs are known to influx and efflux homocysteine. To examine the effect of homocysteine pre-exposure to normal RBCs in the subsequent infection with *Plasmodium* and gametocytogenesis, we performed the following experiment.

Prior to infection, normal RBCs were exposed to 20 μM homocysteine (HCy-RBCs) for four hours and incubated at 37 °C. As a control, normal RBCs (nRBCs) were vehicle-treated and incubated under identical conditions. A concentration of 20 μM of homocysteine was chosen because it is the physiological concentration of homocysteine present in the plasma of malaria patients. Percoll purified late stage trophozoites and schizonts were used to infect these RBCs as described under Methods. Every 24 hrs the parasite culture was replenished with normal cRPMI medium (nMed) or 20 μM homocysteine-containing cRPMI medium (HCy-Med). Four experimental sets were established: Set 1 contained (nRBC) replenished with nMed. Set 2 contained nRBC replenished with HCy-Med. Set 3 contained HCy-RBCs replenished with nMed. Set 4 contained HCy-RBC replenished with HCy-Med. At the end of five days, gametocytes were counted microscopically and gametocytemia was calculated as described under Methods.

**Figure 4:**
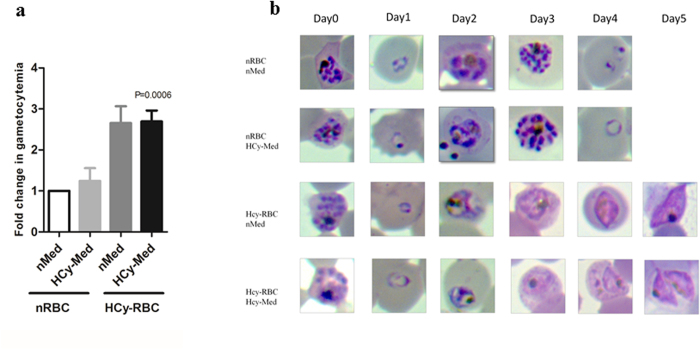
Pre-exposure of erythrocytes to homocysteine enhances gametocytogenesis. Purified late trophozoites/schizonts were added to four sets of RBCs pretreated with vehicle or 20 μM homocysteine for 4 hrs and gametocyte count was taken on Day 5. Medium was replenished every 24 hrs. Set 1: normal RBC (nRBC) with normal medium (nMed) change. Set 2: normal RBC (nRBC) replenished with 20 μM homocysteine containing medium (HCy-Med). Set 3: 20 μM homocysteine pre-treated RBCs (HCy-RBC) in normal medium (nMed). Set 4: 20 μM homocysteine pre-treated RBCs (HCy-RBC) in 20 μM homocysteine containing medium (HCy-Med). (**a**) Figure shows fold change in gametocytogenesis in different sets. Set 1 served as control. Set 2 shows a 1.2-fold increase in gametocytemia. Experimental Sets 3 and 4 containing pre-treated homocysteine RBCs show 2.6 and 2.7-fold increase in gametocytemia, respectively (P < 0.0006, n = 3) (**b**) Representative microscopic images of the above mentioned experimental sets from Day 0 to Day 5. Parasites growing in homocysteine pre-treated RBC clearly showed progression towards gametocytogenesis.

The vehicle treated Set 1 (*nRBC, nMed*) served as the control and showed baseline gametocytemia (Fig. 4a). As expected, Set 2 (*nRBC, HCy-Med*) showed a 1.2 fold increase in gametocytemia with respect to the control (which is similar to the gametocytemia observed in our previous *in vitro* experiment as shown in Fig. 3d). In Set 3 (*HCy-RBC, nMed*), we observed a 2.6-fold increase in gametocytemia with respect to control. In Set 4 (*HCy-RBC, HCy-Med*) the increase in gametocytemia was similar to Set 3 (2.7 folds compared to control). Fig. 4b shows the representative microscopic images depicting commitment towards gametocytogenesis in the four experimental sets. Our results indicate that commitment towards gametocytogenesis is enhanced when parasites invade RBCs that have elevated levels of homocysteine. It strengthens the possibility of homocysteine being a metabolic trigger of gametocytogenesis in malaria patient.

### Phylogenetic Analysis of the TS pathway in parasitic protozoa

Intrigued by our observations in the malaria parasite, we resorted to phylogenetic analysis of the TS pathway across Kingdom Protozoa. A phylogenetic tree was constructed (as described under Methods) taking into account 15 species, comprising 8 species of Phylum Apicomplexa (*P.vivax, P.berghei, P.falciparum* of Order Haemosporidia, *B.bovis, T. annulata* and *T.parva* of Order Piroplasmida and *T.gondii* and *N.caninum* of Order Eucoccidioda), 7 species of Phylum Sarcomastigophora (*T.brucei, T.cruzi, L.major* and *L.braziliensis* of Order Kinetoplastidae, *G.lamblia* of Order Diplomonadida, *T.vaginalis* of Order Trichomonadida and *E.histolytica* of Order Amoebida).

**Figure 5:**
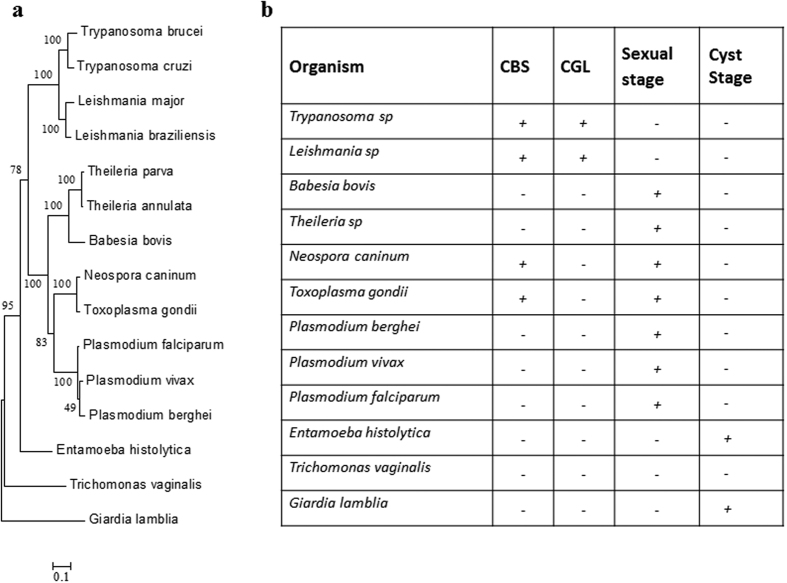
Phylogenetic analysis of the transsulphuration pathway across parasitic protozoa. (**a**) Phylogenetic tree was constructed using the Maximum Likelihood Method algorithm using sequences of the evolutionarily conserved Hsp90. The tree was drawn to scale and branch lengths correspond to number of substitutions per site. (**b**) To examine the evolution of the TS pathway, BLASTp analysis was performed to inspect the presence or absence of CBS and CGL across 11 phylogenetic relatives of *Plasmodium* taking *Saccharomyces cerevisiae* sequences as the template. Table shows the presence (+) or absence (−) of these enzymes in protozoan parasites.

Figure 5 shows the phylogenetic tree constructed using the conserved Hsp90 sequences from the above mentioned parasitic protozoa. The tree depicts the phylogenetic position of each of them. To understand whether a complete TS pathway is operating in these protozoans, we performed a BLASTP search for the enzymes CBS and CGL. The representative protein sequences of CBS (YGR155W) and CGL (YAL012W) of *Saccharomyces cerevisiae* were retrieved from FungiDB. Using these sequences as a query, we looked for the presence of CBS and CGL in the representative protozoans keeping the threshold of E value cutoff as 0.01. The analysis showed that the enzymes CBS and CGL are only present in protozoans namely *Trypanosoma brucei* (Tb11.02.5400, Tb09.211.3330), *Trypanosoma cruzi* (TcCLB.508177.120, TcCLB.510661.250), *Leishmania major* (LmjF17.0252, LmjF35.3230). In case of *Toxoplasma gondii* only CBS (TGME49_259180) was present. The enzymes were found to be missing in *Entamoeba histolytica, Giardia lamblia, Trichomonas vaginalis, Plasmodium* sp, *Theileria* sp, *Babesia* sp.

Based on these results, a table was generated showing the presence (+) or absence (−) of CBS and CGL enzymes in the parasitic protozoa (Fig. 5). It is evident from the table that primitive protozoa like *Giardia* and *Entamoeba*, which are also known to lack the metabolite glutathione^39^,^40^ lack all the enzymes of the TS pathway. Intracellular apicomplexans like *Plasmodium, Theileria* and *Babesia* lack CBS and CGL enzymes of the TS but possess most of the enzymes of the glutathione biosynthesis pathway. As we go up the evolutionary ladder to the extracellular Kinetoplastidae members like *Trypanosoma* and *Leishmania*, it is seen that they have a well-developed TS pathway as well as enzymes involved in glutathione metabolism^13^. It therefore appears that the TS pathway gradually evolved from early eukaryotes like *Giardia* and *Entamoeba* to intracellular Apicomplexans like *Plasmodium* to a fully functional pathway in Kinetoplastids like *Trypanosoma*. From the table, the correlation between the lack of CBS/CGL enzymes, the need for intracellular parasitism and an ability to adapt to sexual stage upon stress is indeed striking.

## Discussion

Protozoan parasites are known to undergo stage transition in response to stressful environment in the host. Amitochondrions like *Giardia* and *Entamoeba* form highly resistant cysts to cope with harsh external environment while intra-erythrocytic parasites like *Plasmodium, Babesia* and *Theileria* undergo sexual stage transition in response to stress^41^. In the current study we have focused on understanding physiological and molecular triggers of gametocytogenesis in malaria.

Gametocytogenesis is a relatively ill explored aspect of malaria biology. Despite advances made in our understanding of specific genes, transcription factors and epigenetic mechanisms involved in the process of gametocytogenesis, the proximal triggers that stimulate the sexual pathway still remain obscure^42^,^43^. We have previously shown that UPR triggers gametocytogenesis in malaria parasite. Using a model redox perturbant, namely DTT we showed that redox imbalance can induce conversion of asexual stage parasites to gametocytes^7^. We have now addressed the physiological relevance of this finding by showing efflux of redox metabolites such as homocysteine from the parasite and its potential role in triggering gametocytogenesis.

In higher eukaryotes, the redox metabolite homocysteine is a component of the TS pathway that converts it to cysteine which further leads into the synthesis of glutathione. Our bioinformatics analysis of genes coding for the enzymes involved in the transsulphuration pathway of malaria parasite revealed the absence of two key enzymes in the biosynthesis of cysteine from homocysteine, namely CBS and CGL. From the leads obtained in our *in-silico* analysis, we validated the disrupted TS pathway in the parasite. We used mass spectrometry to show the absence of a critical metabolic intermediate, cystathionine in *Plasmodium falciparum*. The observation suggested that the parasite is unable to convert homocysteine to cysteine. The truncation in this pathway would therefore result in accumulation of homocysteine in the host RBC and the spent medium. We confirmed this prediction by measuring the levels of homocysteine intracellularly and in the culture supernatant of infected erythrocytes. Our observations provide a possible mechanism that explains a long known fact in malaria biology namely efflux of redox metabolites from *Plasmodium* infected RBC.

To determine how these effluxed redox metabolites influence the redox environment inside the infected RBC and its surroundings, we monitored the GSH/GSSG levels in these compartments. GSH/GSSG redox couple is the predominant redox buffer and is known to define the redox state of a cell. Previous studies have stressed on the importance of GSH/GSSG levels as *de novo* biosynthesis of GSH is critical for parasite survival^23^. However, the values currently available in the literature are variable^2^,^21^,^22^,^23^. There is a discord in the values reported by different groups and a lack of agreement with regard to the form of glutathione being effluxed. We addressed this issue using the highly sensitive approach of mass spectrometry to establish values of GSH and GSSG in the host compartment and the spent medium. Our results suggest that upon infection with the malaria parasite, the host RBC compartment turns mildly oxidizing and there is accumulation of GSSG and efflux of both GSH and GSSG into the medium. Modulation of redox environment of the host seems to be a common theme among various infectious diseases. A decrease in GSH: GSSG ratio is also reported for several viral, bacterial and parasitic infections (Table 1). This change is often linked to a critical biological event involved in the virulence of the pathogen; for example, enhanced pathogenesis and dampening of the host immune response. Our results suggest that *Plasmodium* infection also creates a redox imbalance in its host cell (depicted by a drop in the host GSH: GSSG ratio) due to accumulation and efflux of redox active metabolites.

We also measured levels of other metabolites related in the TS pathway- SAM, SAH, methionine and cysteine. The levels of SAM and SAH were elevated both in the iRBC and iMed compared to nRBC and nMed, respectively. Previous clinical studies which report elevated levels of homocysteine intracellularly and in the plasma of patients, also have reported an increase in the levels of SAM and SAH in the plasma of patients^44^,^45^. *Plasmodium falciparum* depends on an exogenous source of methionine and cysteine for continuous^46^. This can explain why methionine and cysteine levels show a decrease in the iMed.

As mentioned above, homocysteine is abundantly present in the iMed. What would be the effect of the effluxed homocysteine on the parasite? Homocysteine is a known trigger of redox stress and unfolded protein response in eukaryotic cells. We therefore examined if homocysteine could induce redox stress in the parasite. Previously our group had shown that redox stress triggers gametocytogenesis in the malaria parasite. We therefore used gametocytogenesis as a measure of homocysteine induced redox stress. We observed induction of gametocytogenesis on exposure of homocysteine to both *P. falciparum* cultures as well as *P. berghei* infected mice. This result also correlated with increase of AP2-G, the transcription factor believed to be the master regulator of gametocytogenesis^47^,^48^, on exposure of *P. falciparum* cultures to homocysteine. We also found a notable increase in gametocyte numbers when we used RBCs that were pre-loaded with homocysteine concentrations known to be present in plasma of malaria patients^12^. Our result for the first time relates a physiological metabolite with the process of gametocytogenesis and implicates homocysteine to be a potential physiological trigger of gametocytogenesis in malaria. Our study suggests that the truncated transsulphuration pathway in the parasite leads to accumulation of homocysteine in the parasite which is then effluxed out into the spent medium (or plasma of the malaria patient). Indeed, as the asexual cycle number increases, so does the load of homocysteine levels in the circulation of the malaria patient and consequently the number of gametocytes. This is in agreement with the general observation that gametocytemia goes up with cycle numbers of the parasite in the malaria patient^49^.

Erythrocytes are known to be equipped with homocysteine efflux pumps^50^. The homocysteine accumulated in circulation would be taken up by normal erythrocytes based on the local concentration of homocysteine. Any merozoite invading such an erythrocyte would possibly get triggered for sexual conversion. Based on these results it appears possible that depending on circulating levels of homocysteine in the host, the parasite may decide the duration of its asexual cycle in the host; once the homocysteine concentration reaches a particular threshold the parasite senses the redox imbalance created in the host and prepares to exit the host by converting into gametocytes.

Our bioinformatics analysis in Kingdom Protozoa, suggests an evolutionary basis for the absence of the key enzymes in the TS pathway. The analysis shows that primitive protozoan parasites completely lack the TS/glutathione pathway; Apicomplexans have a partially developed pathway while Kinetoplastids have a fully developed one. The trend appears to correlate with evolution of sex in these organisms, with only the apicomplexans which lack the key enzymes in the TS pathway exhibiting sexual stages in their life cycle. It therefore appears possible that the intracellular apicomplexans have refrained from acquiring the missing enzymes in the TS pathway. These parasites seem to have repurposed the truncated pathway and the consequent accumulation of redox metabolites to create a self-regulated switch for sexual conversion during the course of evolution.

## Methods

### Bioinformatics analysis

The sequences of the enzymes of the transsulfuration pathway cystathionine beta synthase (CBS) and cystathionine gamma lyase (CGL) of *Saccharomyces cerevisiae* were collected from NCBI Database. These sequences were used as queries to carry out BLASTP^51^ analysis to identify the homologues present in Protozoa.

The coding sequences of all the genes of the protozoans were obtained from EuPathDB 28. For construction of the phylogenetic tree, Hsp90 sequences were retrieved and analyzed using MEGA 6.06. The sequences used were: *Entamoeba histolytica* EAL47746.1, *Giardia lamblia* BAJ33526.1, *Trichomonas vaginalis* EAY05322.1, *Plasmodium berghei* PBANKA_0805700, *Plasmodium vivax* PVX_087950, *Plasmodium falciparum* PF3D7_0708400, *Toxoplasma gondii* XP_002368278.1, *Neospora caninum* XP_003881046.1, *Babesia bovis* XP_001611554.1, *Theileria annulata* CAI74741.1, *Theileria parva* AAA30132.1, *Leishmania braziliensis* AAY22153.1*, Leishmania major* XP_001685762.1, *Trypanosoma cruzi* AAA30202.1, *Trypanosoma brucei* A44983. Multiple sequence alignment was followed by construction of the phylogenetic tree by maximum likelihood method^52^. The tree with the highest log likelihood (−9058.0494) was shown. The percentage of trees in which the associated taxa clustered together (Bootstrap 1000) is shown next to the branches. The tree is drawn to scale, with branch lengths measured in the number of substitutions per site.

### Cell culturing

#### Plasmodium falciparum

*P. falciparum* 3D7A was cultured in human O+ erythrocytes in complete RPMI 1640 (Sigma Aldrich) medium supplemented with 0.5% (w/v) Albumax II (Invitrogen), 0.2% (w/v) NaHCO3, 0.2% (w/v) Glucose, 200 μM Hypoxanthine and 5 μg/L Gentamycin. Fresh media was added every 24 hrs interval. Cultures were split once the parasitemia reached 5% and were supplemented with fresh RBCs. O+ whole blood was obtained from Red Cross Blood Bank Society, Bangalore.

#### Plasmodium berghei

Mice were maintained and experiments were performed as per the principles, guidelines and methods approved by the Institutional Animal Ethics Committee (IAEC) of the Indian Institute of Science, Bangalore in accordance with Indian National Law on animal care and use (IAEC Ethical approval reference number: CAF/Ethics/269/2012). *P. berghei* ANKA strain was obtained from MR4 and maintained by successive intra-peritoneal injection (10^5^ infected erythrocytes; 200 μl) in Swiss female mice (6–8 weeks old; 22–25 g). Blood collected from tail vein was used to monitor parasitemia using Geimsa stained smears post 48 hrs of infection and was scored microscopically. Mice were considered uninfected if no parasites were observed in 50 fields of view.

### Calculation of half-cell reduction potential of the glutathione couple

The classical Nernst equation was used to calculate the half-cell reduction potential of the GSSG/2GSH couple.

The Nernst equation for reduction potential of the glutathione couple is E_hc_ = E°_hc_ − RT/nF log ([GSH]^2^)/[GSSG] mV; where E_hc_ is the half-cell reduction potential, E°_hc_ is the standard reduction potential, R is the universal gas constant (8.31 JK^−1^mol^−1^), T is the temperature in Kelvin scale, F is the Faraday constant (9.6485 × 104 C mol^−1^), n is the number of electrons transferred in the chemical process^34^.

At 37 °C and pH 7.4,


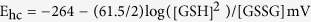


### *In-vitro* gametocytogenesis assay on homocysteine treatment

Parasites cultures were treated with vehicle and 5 mM, 10 mM homocysteine for 1 hr (n = 3). After thorough PBS wash, fresh medium was added to culture and incubated at 37 °C, 5% CO_2_. Percentage gametocytemia (number of gametocytes/total number of parasites * 100) was calculated by counting 5000 RBCs microscopically five days’ post treatment. Data obtained was analyzed using Graphpad Prism 5.0.

### *In vitro* gametocytogenesis assay using homocysteine pretreated RBC

RBCs were exposed to 20 μM homocysteine for four hours (HCy-RBC) and incubated at 37 °C. As a control, normal RBCs (nRBC) were vehicle-treated and incubated under identical conditions. 60% (v/v) Percoll (Sigma) was used to purify late stage trophozoites and schizonts. These parasites were used for infection in the above mentioned RBCs. Every 24 hrs the parasite culture was replenished with normal cRPMI medium (nMed) or 20 μM homocysteine-containing cRPMI medium (HCy-Med). Four experimental sets were used: Set 1 contained (nRBC) replenished with nMed. Set 2 contained nRBC replenished with HCy-Med. Set 3 contained HCy-RBCs replenished with nMed. Set 4 contained HCy-RBC replenished with HCy-Med. These were incubated at 37 °C and 5% CO_2._ The induction of gametocytoegensis was followed from Day 0 to Day 5 and Giemsa stained slides were prepared in each case. 5000 RBCs were counted microscopically and gametocytemia was calculated (n = 3).

### *In-vivo* Gametocytogenesis Assay

Mice were infected as mentioned previously. At 60% parasitemia mice were injected with DTT with the concentrations- which correlated to 2, 20 and 50 mg/Kg body weight respectively (n = 4) and vehicle (PBS) (n = 4). Parasitemia was analysed using tail blood of infected mice post 48 h of infection. Using the same, gametocytemia was scored using Giemsa stained smears considering 5000 RBCs.

Similarly, infected mice were injected directly with homocysteine using concentrations 30 mg/Kg and 60 mg/Kg body weight respectively (n = 4) and vehicle (PBS) (n = 4) and gametocytemia was scored microscopically after 48 hr.

### RNA extraction, cDNA synthesis and qRT-PCR

RNA isolation from *Plasmodium* cells was done using Qiagen Mini Kit as per the manufacturer’s instructions. The concentration and purity of the isolated RNA were evaluated using the Nanodrop Spectrophotometer (Thermo Scientific; 1000). RNA quality was based on the 260/280 values (Nanodrop) and the best quality RNA was used for RT-PCR. Oligo-dT primers were added to RNA and incubated at 65 °C for 15 minutes. Samples were incubated on ice for 2 minutes and reaction mixture of reverse transcription buffer, RNase inhibitor, dNTPs and enzyme was added. Samples were briefly centrifuged and incubated at 42 C for 1 hour for cDNA synthesis.

qRT-PCR amplification was performed on iCycler iQ (Bio-Rad, Hercules, CA) using appropriately diluted cDNA and iQ-SYBR green supermix (Cat. 170-8882). Expression levels of genes were Primer pairs used for the qRT-PCR analysis are listed in Table 3. Expression levels of the genes were normalised against actin, which is a constitutively expressed housekeeping gene.

### Quantification of Cystathionine

Samples were processed at 4 °C unless otherwise stated. Parasitized RBCs were first subjected to 60% (v/v) Percoll purification to enrich metabolically active- late trophozoites and schizonts. iRBCs were then subjected to saponin lysis (final concentration 0.07% of saponin). Post incubation for 10 minutes, the supernatant (saponin lysate) was separated and the parasite pellet (saponin pellet) was washed with PBS. Saponin pellet was then lysed in hypotonic condition using 10 volumes of 20 mM Na-K buffer pH 7.4 followed by three cycles of freeze-thawing in liquid nitrogen. Similarly, saponin lysate was prepared from identical number of nRBC.

*Saccharomyces cerevisiae* lysate was used as a positive control for cystathionine measurement. Towards this, *Saccharomyces cerevisiae* were grown to OD 0.6 and then subjected to glass bead lysis. It was then subjected to high speed spin at 14000 g and the lysate was collected. Yeast lysate, saponin lysate and saponin pellet were subjected to 10 (v/v) 10% TCA precipitation and incubated on ice for 30 mins. This was followed by a high speed spin at 14000 g. Thereafter the supernatant from the above-mentioned samples were independently analysed by LC-MS/MS for cystathionine. For HPLC methods and MS parameters please refer to supplementary methods^53^. Mass spectrometric analysis was performed with electrospray ionization (ESI) operated in multiple reactions monitoring (MRM) in positive mode. The MRM for cystathionine (223 > 134)^53^ was performed by collision energy optimized for each transition. Sample recoveries were optimized to 98–105%.

### Preparing samples for measuring efflux of metabolites in spent medium

Equal number of normal RBCs and parasitized RBCs (parasitemia ranging between 3–4%) were incubated at 5% haematocrit as per previous conditions. After 24 hours, the spent medium was processed for LC-MS and was analyzed for the metabolites as elaborated below. Correction for parasitemia was performed as described elsewhere^22^.

### Preparing samples for quantification of metabolites in erythrocytes

Parasite cultures maintained at 5% haematocrit were allowed to grow to 5% parasitemia. 60% (v/v) Percoll purification was performed as described above. Identical number of nRBC and iRBC were subjected to saponin lysis as previously mentioned. The saponin lysate was processed for LC-MS as described and analysed for GSH, GSSG and homocysteine, SAM, SAH, methionine and cysteine as elaborated below.

### Processing of samples and LC-MS detection of metabolites

#### GS-NEM, GSSG

Samples were treated to a final concentration of 20 mM N-ethyl maleimide (NEM) to alkylate GSH^54^. After incubation for 30 minutes, 10 v/v 10% TCA was added and incubated for 30 minutes. Post incubation samples were spun at 14000 g for 10 minutes. The supernatant obtained was then subjected to HPLC-MS analysis. The MRM for GS-NEM (m/z 433 > 304)^54^ and GSSG (m/z 613.2 > 355)^55^.

#### HCy-NEM

Samples were treated to a final concentration of 10 mM DTT for 30 minutes^56^ followed by alkylation with 20 mM NEM for 30 minutes to obtain total homocysteine concentration. After incubation 10 volumes of 10% TCA was added and incubated for 30 minutes. Thereafter, samples were spun at 14000 g for 10 minutes. Supernatant collected was then subjected for HPLC-MS. The MRM for Hcy-NEM (m/z 261 > 215).

#### Cys-NEM

Samples were treated to a final concentration of 20 mM N-ethyl maleimide (NEM) to alkylate Cys. After incubation for 30 minutes, 10 v/v 10% TCA was added and incubated for 30 minutes followed by centrifugation 14000 g for 10 minutes. The supernatant obtained was then subjected to HPLC-MS analysis. The MRM for Cys-NEM (m/z 247.1 > 158.1).

#### Met, SAH

10 volumes of 10% TCA was added and incubated for 30 minutes. Thereafter, samples were spun at 14000 g for 10 minutes. Supernatant collected was then subjected to HPLC-MS. The MRM for Met (m/z 150.1 > 104.2) and SAH (m/z 385.1 > 136).

#### SAM

10 volumes of 0.2% formic acid in methanol was added to the sample and incubated for 30 minutes followed by a spin at 14000 g for 10 minutes. The collected supernatant was subjected to HPLC-MS analysis. The MRM for SAM (m/z 399.1 > 250.1).

Quantification of metabolites was carried out using HPLC-MS/MS using Agilent 1200 series HPLC coupled with tandem Agilent 6460 QQQ mass spectrometer. For detailed methods and MS parameters please refer Supplementary File 2.

### Statistical Analysis

Results were reported as Mean ± S.E.M. Grouped data was statistically analyzed using one-way ANOVA. For paired comparisons, two-tailed P-test was used. All analysis was done using GraphPad Prism 5.

## Additional Information

**How to cite this article**: Beri, D. *et al*. A disrupted transsulphuration pathway results in accumulation of redox metabolites and induction of gametocytogenesis in malaria. *Sci. Rep.* **7**, 40213; doi: 10.1038/srep40213 (2017).

**Publisher's note:** Springer Nature remains neutral with regard to jurisdictional claims in published maps and institutional affiliations.

## Supplementary Material

Supplementary Information

## Figures and Tables

**Table 1:** Table compiling data from previous studies showing fold decrease in GSH: GSSG ratio in intracellular pathogens (virus, bacteria and protozoa) and its biological relevance.

Organism	Infection	Fold decrease in GSH/GSSG ratio	Biological Relevance
**Virus**	H5N1	2.1	Enhances viral replication and expression of viral proteins^33^
	Respiratory syncytial virus (RSV)	2.6	Perpetuation and amplification of inflammatory response, enhanced viral pathogenesis^29^
	HIV	3.7	HIV-1 persistence and reactivation^25^,^26^,^31^
	Nipah Virus	1.6	ROS activation, DNA damage of host leading to increase in viral pathogenesis^27^
	Hepatitis C Virus	2.3	Replication, progression and severity of HCV infection^30^
	Dengue Virus	1.6	Viral replication^32^
**Bacteria**	*Mycobacterium tuberculosis*	2.5	Increased pathogenesis, alleviates the effect of host immune response^24^
	*Mycoplasma pneumoniae*	2.7	Enhanced pathogenesis^57^
**Protozoa**	*Plasmodium falciparum*	3.7	Redox perturbation^2^,^21^,^22^ Redox stress as a physiological trigger of gametocytogenesis (**Current Study**)
	*Theileria annulata*	2.5	HIF-1α Stabilization, Increased glucose uptake, parasite induced transformation^28^

**Table 2:** Levels of metabolites of the transsulphuration pathway in the normal red blood cells (nRBC), *Plasmodium falciparum* infected red blood cells (iRBC), spent medium of normal cells (nMed) and spent medium of *Plasmodium falciparum* infected red blood cells (iMed).

Metabolite	nRBC (nmoles/10^10^ cells)	iRBC (nmoles/10^10^ cells)	nMed (nmoles/10^10^ cells)	iMed (nmoles/10^10^ cells)
**Methionine**	142 ± 0.7335	14.94 ± 1.048	1867 ± 236.7	1476 ± 265.5
**SAM**	4.643 ± 0.4742	8.992 ± 0.8553	0.08713 ± 0.003494	1.048 ± 0.01958
**SAH**	5.937 ± 0.6817	6.783 ± 0.8332	0.2006 ± 0.02488	0.9383 ± 0.1205
**Homocysteine**	8.064 ± 2.781	17.01 ± 4.431	14.06 ± 1.952	1560 ± 62.16
**Cysteine**	2341 ± 23.93	2652 ± 50.60	2592 ± 33.28	1064 ± 279.2
**GSH**	1304 ± 28.15	729 ± 33.45	0.3731 ± 0.0516	103.7 ± 18.15
**GSSG**	27.76 ± 1.607	53 ± 0.5774	2.657 ± 0.1997	162.4 ± 39.82

**Table 3:** Details of q-RT PCR primers used in the study.

Gene ID		Primer Sequence
**PfActin (PFL2215w)**	Fwd	GAGATGATGCACCTCGTTCC
	Rev	CACTGGGTGTTCTTCTGGAG
**PfAP2-G (PFL1085w)**	Fwd	TTCCAACACGAAAGGAGAGC
	Rev	TGCATGCTCTCTTCCCATTC
**PbAP2-G (PBANKA_143750)**	Fwd	AGATCAGGGAAAATGCGATG
	Rev	TATGCACACGATTCCCGTAA
**PbActin (PBANKA_145930)**	Fwd	CAGAAGCCCCATTAAATCCA
	Rev	AGTCCCTTCCAGCCAAATCT
