# Supplementary material for: A disrupted transsulphuration pathway results in accumulation of redox metabolites and induction of gametocytogenesis in malaria
Source: Sci Rep. 2017 Jan 16;7:40213. doi: 10.1038/srep40213 (PMC5238400; doi:10.1038/srep40213)
Supplement: Supplementary Information [file srep40213-s1.pdf]

A disrupted transsulphuration pathway results in accumulation of redox metabolites and induction of gametocytogenesis in malaria

Divya Beri[#], Balu Balan[#] Shweta Chaubey, Suraj Subramaniam, Bachu Surendra, Utpal Tatu*

#These authors contributed equally to this work *Corresponding author

Divya Beri: Department of Biochemistry, Indian Institute of Science, Bangalore – 560012, India
Email: divya@biochem.iisc.ernet.in

Balu Balan: Department of Biochemistry, Indian Institute of Science, Bangalore – 560012, India
Email: balubalan1410@gmail.com

Shweta Chaubey: Department of Biochemistry, Indian Institute of Science, Bangalore – 560012, India
Email: shwetacbiochem@gmail.com

Suraj Subramaniam: Department of Biochemistry, Indian Institute of Science, Bangalore – 560012, India
Email: suraj12000@gmail.com

Bachu Surendra: Department of Biochemistry, Indian Institute of Science, Bangalore – 560012, India
Email: surendrabpharm49@gmail.com

Utpal Tatu*: (Professor) Corresponding author
Department of Biochemistry, Indian Institute of Science, Bangalore - 560012, India.
Telephone: (080) 22932823; E-mail: tatu@biochem.iisc.ernet.in

Supplementary Figure 1

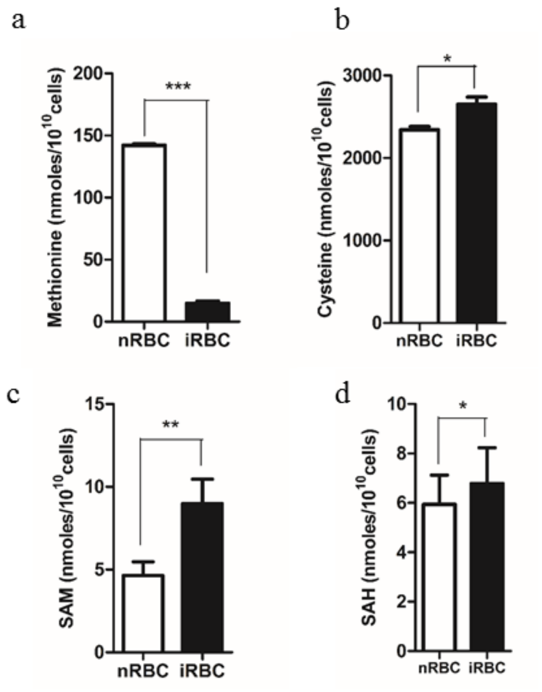

Fig S1: Measurement of metabolites in normal and infected red blood cells. Levels of metabolites were measured in normal red blood cells (nRBC) and *Plasmodium falciparum* infected red blood cells (iRBC). (a) a 9.5-fold decrease in level of methionine in iRBC compared to nRBC (P value <0.0001; n=3). (b) A 1.13-fold increase in level of cysteine in iRBC compared to nRBC (P value = 0.0320; n=3). (c) A 1.93-fold increase in level of SAM in iRBC compared to nRBC (P value = 0.0084; n=3). (d) A 1.12-fold increase in level of SAH in iRBC compared to nRBC (P value = 0.0461; n=3).

Supplementary Figure 2

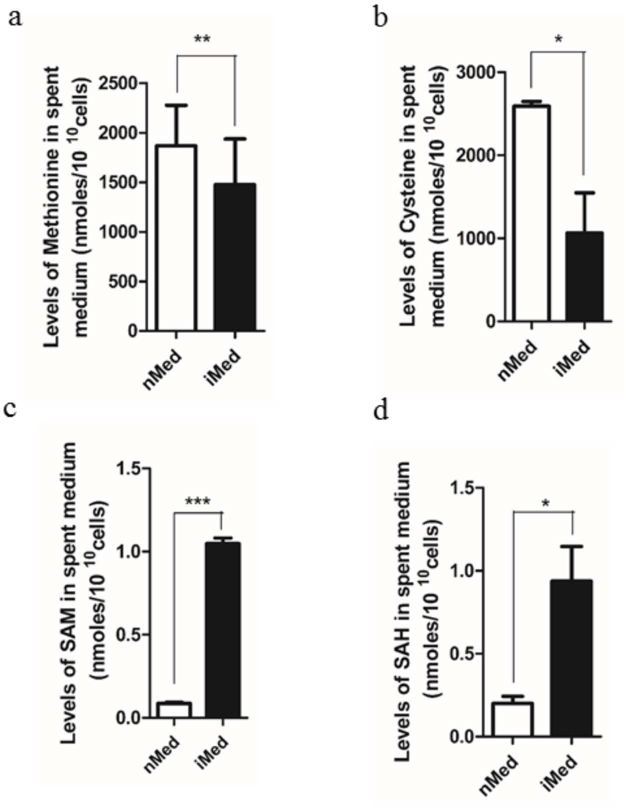

Fig S2: Measurement of metabolites in spent medium of normal and infected red blood cells. Levels of metabolites were measured in spent medium of normal RBCs (nMed) and *Plasmodium falciparum*- infected red blood cells (iMed). (a) A 1.3-fold decrease in level of Methionine in spent medium of infected erythrocytes compared to normal erythrocytes (P value 0.0099; n=3). (b) A 2.5-fold decrease in the level of cysteine in spent medium of infected erythrocytes (iMed) compared to normal erythrocytes (nMed) (P value = 0.0364; n=3). (c) A 12-fold increase in the level of SAM in spent medium of infected erythrocytes (iMed) compared to normal erythrocytes (nMed) (P value = 0.0003; n=3). (d) A 4.7-fold increase in the level of SAH in spent medium of infected erythrocytes (iMed) compared to normal erythrocytes (nMed) (P value = 0.0175; n=3).

Supplementary File 2

LC-MS/MS Methods for GS-NEM,GSSG,HCy-NEM, Cystathionine, Methionine, SAM, SAH and L-Cys-NEM.

GS-NEM: (433.1>304)

Column:	Zorbax Eclipse Plus C18 (4.6x 250 mm; 5 micron particle size)
Mobile phase:	Methanol: water 0.3% formic acid (40:60) v/v
Flow rate:	1ml/min
Runtime:	5 mins
Retention time:	2.67 ± 0.3 mins
Extraction:	100 µl sample + 900 µl 10% TCA
Linearity range:	10, 50, 100, 200, 500, 1000, 2000, 5000 ppb

GSSG (613>355)

Column:	Zorbax Eclipse Plus C18 (4.6x 250 mm; 5 micron particle size)
Mobile phase:	Methanol: water 0.3% formic acid (5:95) v/v
Flow rate:	1ml/min
Runtime:	7 mins
Retention time:	5.95 ± 0.3 mins
Extraction:	100 µl sample + 900 µl 10% TCA
Linearity range:	5, 10, 50, 100, 300, 500 ppb

Cystathionine (223>134)

Column:	Thermo hypersil gold HILIC (4.6x150mm; 5-micron particle size)
Mobile phase:	Acetonitrile: 0.1% Formic acid in Water (20:80) v/v
Flow rate:	0.6 ml/min
Runtime:	4 mins
Retention time:	2.82 ± 0.3 mins
Extraction:	100 µl sample + 900 µl 10% TCA
Linearity range:	0.3, 0.5, 1, 3, 5, 10, 30, 50 ppb

HCY-NEM (261>215)

Column:	Zorbax Eclipse Plus C18 (4.6x 250 mm; 5-micron particle size)
Mobile phase:	Methanol: water 0.3% formic acid (20:80) v/v
Flow rate:	0.8 ml/min
Runtime:	7 mins
Retention time:	6.22 ± 0.3 mins
Extraction:	100 µl sample + 900 µl 0.2% formic acid in Methanol
Linearity range:	1, 2, 10, 20, 50, 100, 200, 500 ppb

Methionine: (150.1>104.2)

Column:	Zorbax Eclipse Plus C18 (4.6x 250 mm; 5-micron particle size)
Mobile phase:	Methanol: 0.3% formic acid in Water (5:95) v/v
Flow rate:	0.8 ml/min
Runtime:	8 mins
Retention time:	6.742 ± 0.3 mins
Extraction:	50 µl sample + 450 µl 10% TCA
Linearity range:	20, 50, 100, 200, 500, 1000 ppb

SAM: (399.1> 250.1)

Column:	Zorbax Eclipse Plus C18 (4.6x 250 mm; 5-micron particle size)
Mobile phase:	Methanol: 0.3% formic acid in Water (55:45) v/v
Flow rate:	0.8 ml/min
Runtime:	4 mins
Retention time:	2.573 ± 0.3 mins
Extraction:	50 µl Sample + 950 µl (0.2% formic acid in Methanol)
Linearity range:	0.5, 1, 2.5, 5, 10, 25, 50, 100 ppb

SAH: (385.1>136)

Column:	Zorbax Eclipse Plus C18 (4.6x 250 mm; 5-micron particle size)
Mobile phase:	Methanol: 0.3% formic acid in Water (5:95) v/v
Flow rate:	0.8 ml/min
Runtime:	8 mins
Retention time:	7.272 ± 0.3 mins
Extraction:	50 µl sample + 450 µl 10% TCA
Linearity range:	0.5, 1, 2.5, 5, 10, 25, 50, 100 ppb

LCYS -NEM: (247.1> 158.1)	
Column:	Zorbax Eclipse Plus C18 (4.6x 250 mm; 5-micron particle size)
Mobile phase:	Methanol: 0.3% formic acid in Water (15:85) v/v
Flow rate:	0.8 ml/min
Runtime:	7 mins
Retention time:	5.937 ± 0.3 mins
Extraction:	100 µl Sample + 900 µl (10% TCA)
Linearity range:	1, 5, 20, 50, 100, 200 ppb
